# The optimal post-eclosion interval while estimating the post-mortem interval based on an empty puparium

**DOI:** 10.1007/s12024-020-00328-y

**Published:** 2020-11-09

**Authors:** Jędrzej Wydra, Szymon Matuszewski

**Affiliations:** Laboratory of Criminalistics, Adam Mickiewicz University, Św. Marcin 90, 61-809, Poznań, Poland

**Keywords:** Forensic entomology, Post-mortem interval, Empty puparium, Uncertainty, Simulation

## Abstract

The puparium is the hardened exoskeleton of the last larval instar of a fly, inside which a prepupa, a pupa and a pharate adult fly successively develop. Empty puparia are frequently collected at death scenes, especially in cases with a long post mortem interval (PMI). Although we are not able to estimate the interval between the eclosion of an adult fly and the collection of an empty puparium (i.e. the post-eclosion interval (PEI)), empty puparia may still provide valuable evidence about the minimum PMI. However, because of the unknown PEI, it is impossible to determine the time when the fly emerged, and thus when the retrospective calculation of the minimum PMI should start. In this study, the estimation of PMI (or minimum PMI) for empty puparia of *Protophormia terraenovae* Rob.-Desv. (Calliphoridae) and *Stearibia nigriceps* Meig. (Piophilidae) was simulated, to gain insight into the changes in estimates, when different PEIs and different temperature conditions were assumed. The simulations showed that the PEI (in a range of 0–90 days) had no effect on the PMI (or minimum PMI) when the puparium was collected in winter or early spring (December–April). In late spring, summer, or autumn (May–November) the PMI (or minimum PMI) increased with the PEI. The increase in PMI was large in the summer months, and surprisingly small in the autumn months, frequently smaller than the PEI used in the estimation. The shortest PMI was always obtained with a PEI of 0, indicating that the true minimum PMI is always estimated using a PEI of 0. When the puparium was collected during spring, simulations indicated that oviposition had occurred in the previous year, while in summer the previous-year oviposition has been indicated by the simulations only when longer PEIs had been assumed. These findings should guide estimation of the PMI (or minimum PMI) based on an empty puparium.

**Supplementary Information:**

The online version contains supplementary material available at (10.1007/s12024-020-00328-y).

## Introduction

The post-mortem interval (PMI) can be estimated from insect evidence [1–6]. Flies are frequently used for this purpose, and their larvae or puparia are the pieces of insect evidence with the highest incidence on a death scene [7–11]. The puparium is a hardened exoskeleton of the last larval instar of a fly, inside which a prepupa, pupa and pharate adult fly successively develop [12, 13]. Upon development, the adult fly emerges from the puparium. The resulting empty puparial case can remain at the death scene for a very long time [14]. Empty puparia are frequently collected in forensic contexts, especially in cases with a long PMI [2, 15]. However, estimating the PMI based on such evidence encounters serious difficulties.

First, the interval between fly eclosion and the collection of an empty puparium, called the post-eclosion interval (PEI) [2] is usually unknown. Puparia are very durable; the PEI may be long enough to make the minimum PMI much less accurate. Several papers addressed the issue of PEI estimation and mostly proposed that the analysis of the puparium chemistry might be useful in helping determine the PEI [16–21]. Unfortunately, none of these propositions have currently resulted in the development a method that would be useful in routine forensic investigations and we are not convinced it will be possible to estimate the PEI in the near future.

Although we are not able to estimate the PEI, an empty puparium can still provide valuable evidence in terms of the minimum PMI. However, because of the unknown PEI, it is impossible to determine when the fly emerged and the premature development was completed. Insect age is estimated retrospectively using the temperatures that prevailed before the end of development [22–25]. If we do not know when the development was completed, we usually do not know what temperatures to use and thus when to start retrospective thermal summation. In practice, the age at eclosion was estimated using average temperatures in the months preceding collection of the puparium, thus providing alternative estimations for different months [2]. Another solution was to use a standard thermal summation procedure and to start calculations at the time of the evidence collection [1]. However, the effects of these protocols on the age or the PMI estimation were not investigated. In addition, the difficulty in choosing the time to start calculations has wider implications. The age estimation is sometimes supplemented with the pre-appearance interval (PAI) estimation [2]. PAI also requires a specific moment to start calculations [26, 27]. When estimating the PAI for an empty puparium, this is a moment at which development began (oviposition or larviposition), and as stated above it cannot usually be reliably determined. Therefore, the estimation of PAI is limited in a similar way as age estimation.

In this study, we simulated the age and PAI estimation for empty puparium, to gain insight into changes in PMI or minimum PMI when different PEIs and different temperature conditions were assumed. The simulations were performed for *Protophormia terraenovae* Rob.-Desv. (Calliphoridae) and *Stearibia nigriceps* Meig. (Piophilidae), using temperature data from around the year and different estimation scenarios.

## Materials and methods

**Fig. 1:**
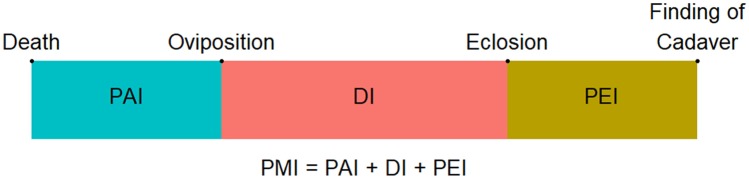
The post-mortem interval (PMI) definition when using an empty puparium for the estimation. PAI – the pre-appearance interval; DI – the development interval; PEI – the post-eclosion interval

In the case of an empty puparium, the post-mortem interval (PMI) consists of three intervals: the pre-appearance interval (PAI), the development interval (DI) and the post-eclosion interval (PEI). There are four important points on this timeline: death, oviposition, eclosion and the finding of a cadaver (i.e. collection of the puparium; Fig. 1).

**Fig. 2:**
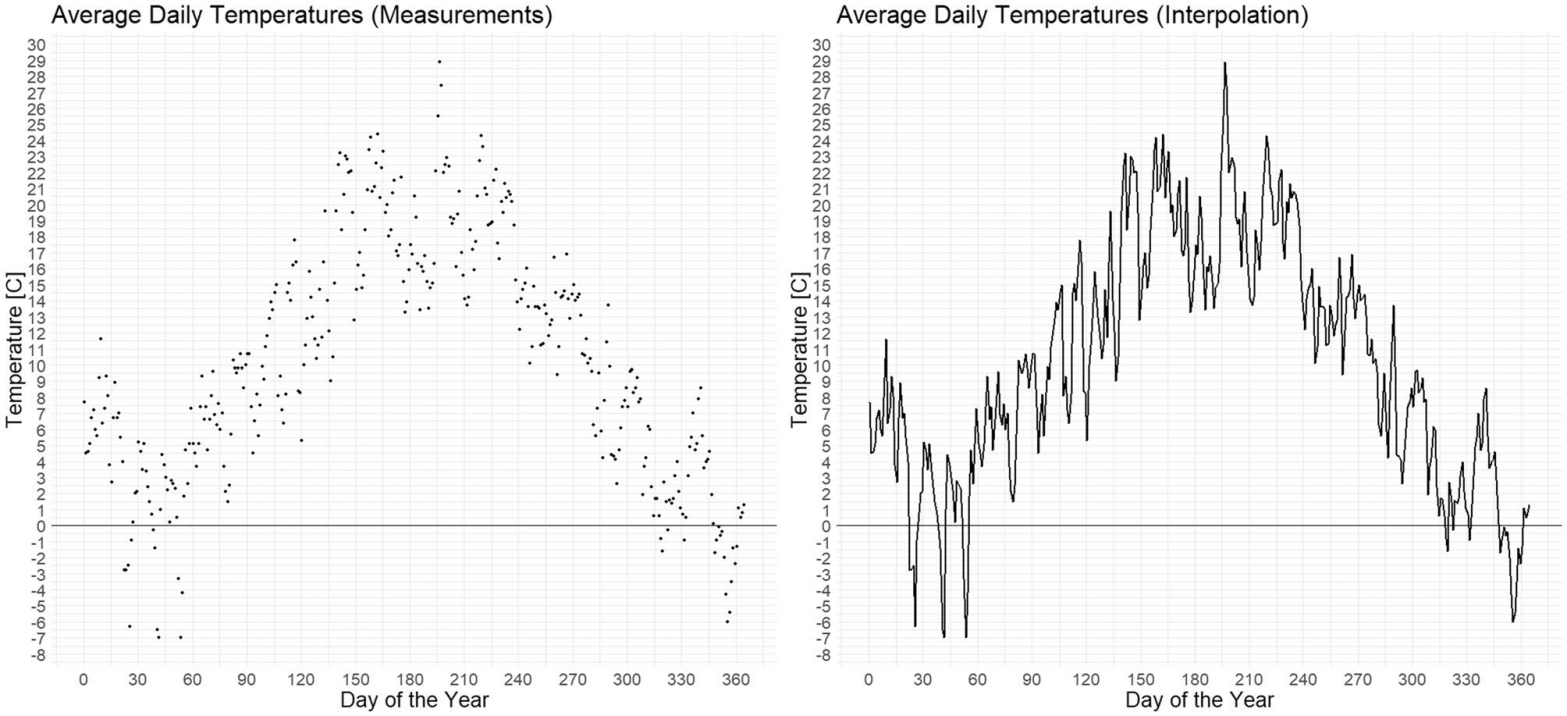
The temperature data used in the simulations, spanning the entirety of 2007

The estimation of PMI was simulated for each day of the year. We used the average daily ambient air temperatures from the local weather station (Marcelin, Poznań, Western Poland) for 2007 and, if necessary, 2006 (Fig. 2). The PMI (PAI + DI + PEI) was estimated for *S. nigriceps* and the minimum PMI (DI + PEI) for *P. terraenovae*. Ninety-one PEIs were used, from 0 to 90 days.

The DI was estimated using the thermal summation method [28–31]. A lower developmental threshold (${t}_{0}$) of $6.4 ^\circ{\rm C}$ and a thermal summation value for the eclosion ($K$) of $434$ degree-days [28] were used for *S. nigriceps*; ${t}_{0}$ of $8.9 ^\circ{\rm C}$ and $K$ of $240.2$ degree-days were used for *P. terraenovae* [32].

The PAI was estimated for *S. nigriceps* only*.* We used the temperature method [27] and the model developed by Matuszewski et al. [33]. The first predictor temperature was the average temperature for a 7-day period before the estimated day of oviposition. The estimation was iterated using the corrected predictor temperature (the average temperature for the PAI estimated in the previous step), as described by Matuszewski and Mądra-Bielewicz [27]. When we started to estimate DI in spring (May or June), it was usually necessary to use the low temperatures of early spring or winter. As a consequence, the PAI became unrealistically high [27]. The PAI estimation was biologically invalid in such cases, because *S. nigriceps* does not oviposit in winter. Accordingly, the estimation procedure was modified for the spring months. When the oviposition was estimated to occur in winter, we added an extra interval to move it beyond the cold season. As a result, the PAI was estimated using warmer autumn temperatures. The length of this extra interval depended on the estimated day of oviposition.

The simulations were implemented in *R* (version 3.6.3). The number of degree-days was calculated for each day across 2007 and 2006. To obtain the DI, the degree-days were accumulated until $K$ for the species was reached. The DI was calculated for each day of 2007 and then these values were adjusted to include 91 PEIs. The procedure was the same for both species, $K$ being the only difference. Additionally, in the case of *S. nigriceps* we calculated PAI. We obtained 33 215 PMI and minimum PMI simulations (365 days * 91 PEIs). Datasets are provided in the supplementarymaterial.

## Results

**Fig. 3:**
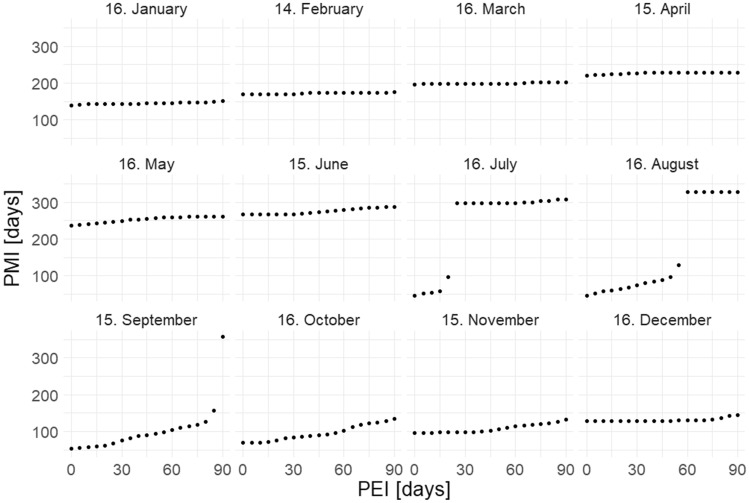
Simulations of the post-mortem interval (PMI) for an empty puparium of *Stearibia nigriceps*, plotted against the assumed post-eclosion interval (PEI). Simulations for a single day (the middle of a month) are shown in each month. Every fifth data point is presented

**Fig. 4:**
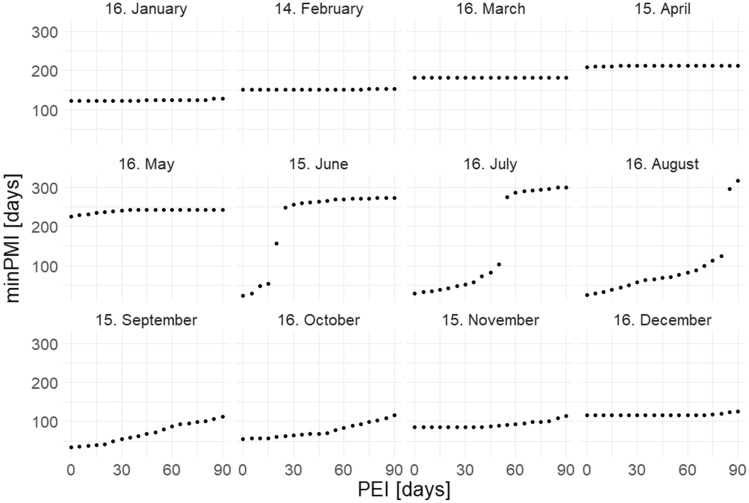
Simulations of the minimum post-mortem interval (minimum PMI) for an empty puparium of *Protophormia terraenovae*, plotted against the assumed post-eclosion interval (PEI). Simulations for a single day (the middle of a month) are shown in each month. Every fifth data point is presented

Similar patterns were found for both species. Changes in PEI had no effect on the PMI (or minimum PMI) in winter and early spring (December–April, Fig. 3, 4). In late spring, summer and autumn (May–November) the PMI (or minimum PMI) increased with PEI (Fig. 3, 4). If a particular PEI was used in summer, the increase in PMI was very rapid (Fig. 3, 4). In the case of the autumn months, the increase in PMI was small, frequently smaller than the currently used PEI (Fig. 3, 4).

**Fig. 5:**
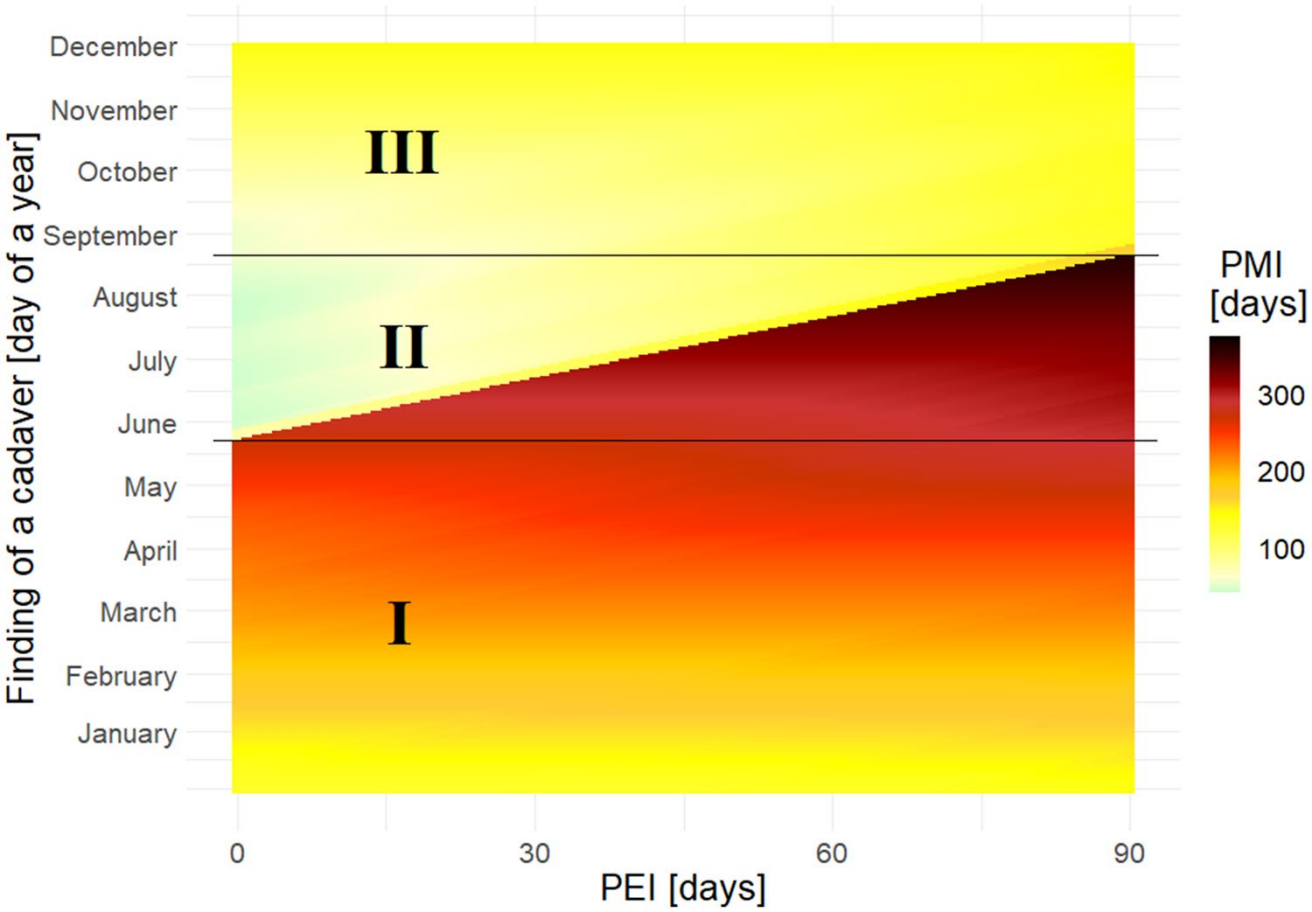
Simulations of the post-mortem interval (PMI) across the whole year for an empty puparium of *Stearibia nigriceps* and assuming different post-eclosion intervals (PEI). Horizontal lines divide the year into three periods, which differ in the effect of PEI on PMI

**Fig. 6:**
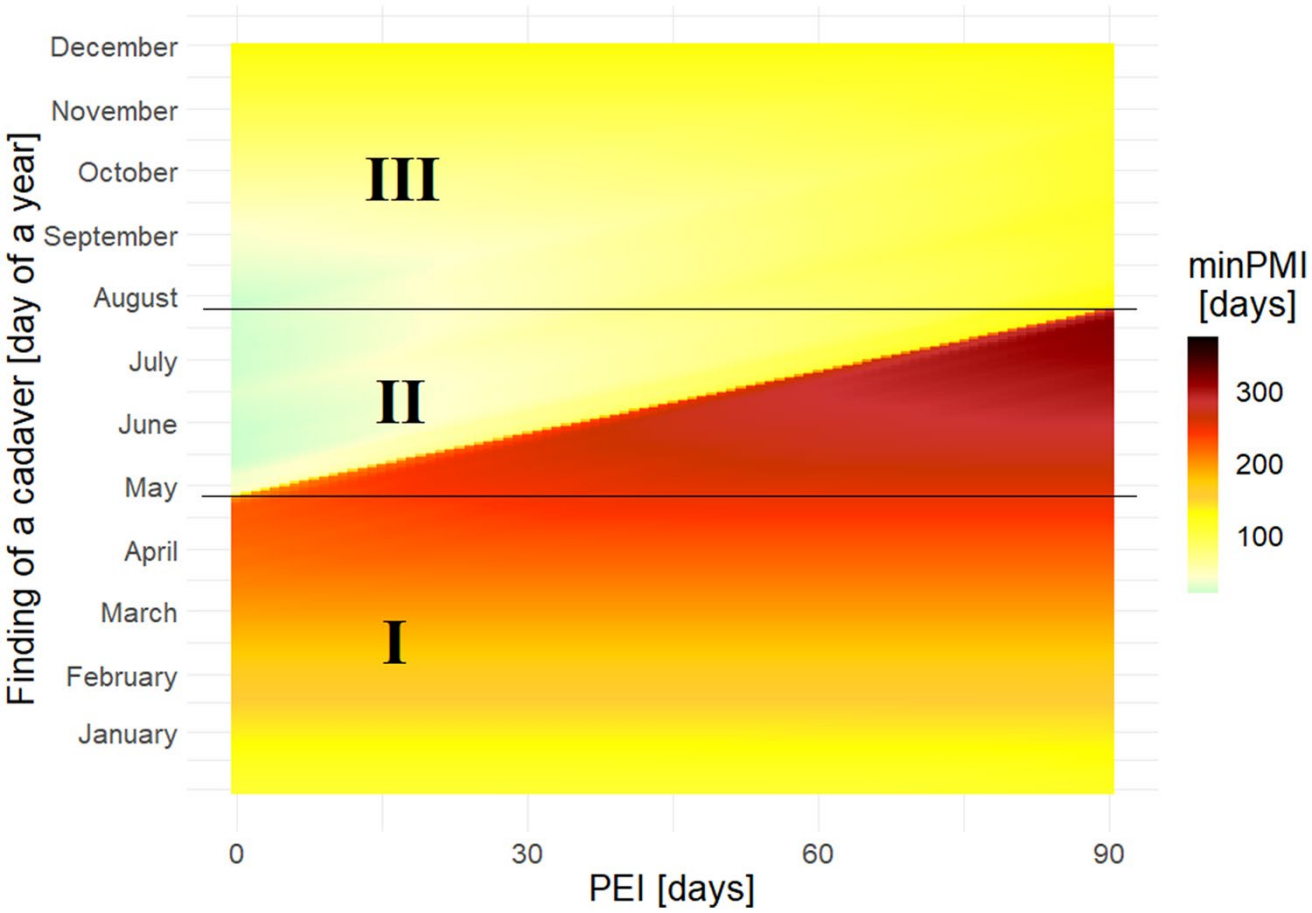
Simulations of the minimum post-mortem interval (minPMI) across the whole year for an empty puparium of *Protophormia terraenovae* and assuming different post-eclosion intervals (PEI). Horizontal lines divide the year into three periods, which differ in the effect of PEI on PMI

The simulations showed that the shortest PMI was always obtained with a PEI of 0 (Fig. 3–4, and a mathematical proof in the Supplementary Material). Simulations plotted for the whole year (Fig. 5–6) revealed three distinct periods. The first one extended from January until about mid-June for *S. nigriceps* (I, Fig. 5) and until about the end of May for *P. terraenovae* (I, Fig. 6). The PMI was very long for these months, indicating that the oviposition had to occur in the previous year. The second period extended from about mid-June until about mid-September for *S. nigriceps* (II, Fig. 5) and from about the end of May until about the end of August for *P. terraenovae* (II, Fig. 6). During this period very long PMIs were obtained, but only for certain PEIs. The third period extended over the rest of the year (III, Fig. 5–6). The PMIs were rather moderate during this part of the year and from September until November they were only marginally affected by the PEI.

## Discussion

A lack of relation between the PEI and the PMI (or minimum PMI) in the winter and early spring months is a consequence of the very low temperatures in these months, which are usually considerably below the developmental threshold of the species (Fig. 2). Whatever the PEI was (in a range of 0–90 days), it simply coincided with a period of no-development, and for this reason, it had no effect on the PMI (or minimum PMI).

The rapid increase in PMI at certain levels of PEI, recorded in summer, was an indirect consequence of the temperature differences between spring and summer (Fig. 2). For some PEIs (e.g. 60 days or more for the August scenario and *S. nigriceps*, Fig. 3), the age at eclosion was estimated using lower spring temperatures instead of higher summer temperatures, resulting in a higher estimated age. The PMI was much longer in such cases, due to the inclusion of the PEI and higher age.

The small increase in PMI recorded in autumn was also an effect of the temperature differences; in this case between autumn and summer (Fig. 2). The age at eclosion in autumn was estimated for most PEIs using higher summer temperatures, resulting in lower PMIs. The increase in PMI following the inclusion of PEI was partially offset by a decrease in the insect age.

Our simulations revealed patterns that are important for PMI estimation based on an empty puparium. Most importantly, a PEI of 0 always yielded the shortest PMI. This pattern was present in the case of both species and all estimation scenarios. Although in autumn an increase in PMI with an increase in PEI was usually partially compensated by a decrease in the fly age, the age decrease was never greater than or equal to the PEI. Therefore, the current results conclusively demonstrate that the true minimum PMI is always estimated using a PEI of 0. Accordingly, forensic entomologists should always start their retrospective PMI estimations from the moment of collection of an empty puparium (i.e. assuming 0 for the PEI) unless there are good reasons to use a longer PEI. Even if there are such reasons, experts should be cautious in this regard, as such decisions make the PMI much higher (especially in summer), and therefore there is a risk of overestimation of PMI.

The simulations carried out for the whole year revealed three periods that differ in the effect of the PEI on the PMI. The first one, which extended from January until about May or June (depending on the species), always had very long PMI estimates, which indicated that the oviposition occurred in the previous year. Such patterns depend on temperature, and they can change at different temperatures. However, the presence of a long period during which an empty puparium indicates that oviposition occurred in the previous year is highly likely; for different species only the length of this period may change. Accordingly, if the temperature conditions are similar or colder than conditions in this study, the possibility of oviposition occurring in the previous year must be considered for cases in which an empty puparium is collected in spring (and of course winter). The second period, which covered late spring and summer, had very long PMIs only under long PEIs. These simulations show that the PEI cannot be longer than a certain value; otherwise oviposition having occurred in the previous year would have to be considered. If an empty puparium of *S. nigriceps* was found, for example in mid-July, the PEI cannot be longer than 24 days, unless the oviposition occurred in the previous year. Indeed, the PEI had the largest impact on the PMI during the summer months*.* Therefore, extreme caution is needed when choosing a PEI greater than 0 at this time. For the third period, which largely extended into autumn, there were moderate PMI estimates, and they always indicated that oviposition occurred in the current year.

Although the patterns for *S. nigriceps* and P. *terraenovae* were very similar, there were some differences. The period that indicated the previous-year oviposition was about one month longer for empty puparia of *S. nigriceps*. *S. nigriceps* colonizes cadavers later than *P. terraenovae* and its premature development lasts longer [28, 32, 34, 35]. As a consequence, *S. nigriceps* needs more time to complete its development after winter. We hypothesize that this pattern is characteristic for all the fly species that colonize cadavers late in decomposition.

### Key points

1. Empty puparia are frequently collected at death scenes and may provide valuable evidence about the post-mortem interval (PMI).
2. We simulated the estimation of PMI for empty puparia of *Protophormia terraenovae* (Calliphoridae) and *Stearibia nigriceps* (Piophilidae), assuming different post-eclosion intervals (PEI) and various temperature conditions.
3. The study revealed that PEI (of 0–90 days) had no effect on PMI in winter or early spring, whereas for the rest of the year PMI became larger with PEI.
4. The shortest PMI was always obtained with a PEI of 0, demonstrating that minimum PMI is always estimated using a PEI of 0.

## Electronic supplementary material

Below is the link to the electronic supplementary material.

Supplementary file (DOCX 19.7 kb)

dataset I Protophormia terraenovae (XLSX 722 kb)

dataset II Stearibia nigriceps (XLSX 855 kb)
