# Supplementary material for: The optimal post-eclosion interval while estimating the post-mortem interval based on an empty puparium
Source: Forensic Sci Med Pathol. 2020 Nov 9;17(2):192–8. doi: 10.1007/s12024-020-00328-y (PMC8119268; doi:10.1007/s12024-020-00328-y)
Supplement: Supplementary file 1 — Supplementary file (DOCX 19.7 kb) [file 12024_2020_328_MOESM1_ESM.docx]

**Supplementary Material**

# **Appendix**

This appendix contains a mathematical demonstration that the shortest minimum $PMI$ ($minPMI$) is always obtained with a $PEI$ of 0.

The formula for degree-days is as follows:

$$DD=\left( T-t_{0} \right)d$$

where $DD$ denotes the number of degree-days, $T$ denotes the average temperature, $t_{0}$ denotes lower developmental threshold and $d$ denotes the number of days. When $T<t_{0}$, then $DD=0$.

The thermal summation method [[28-30](#_ENREF_28)] assumes accumulating degree-days backward to some predefined value, e.g. degree-days needed for insect eclosion ($K$). For each day we used average daily temperatures and the formula:

$${DD}_{i}=\left( T_{i}-t_{0} \right)$$

where ${DD}_{i}$ is the number of degree-days in $i$-th day and $T_{i}$ is the average daily temperature of the $i$-th day.

The development interval ($DI$) is deduced using the equation:

$$K= {DD}_{1}+{DD}_{2}+{DD}_{3}+\ldots+ {DD}_{n}=\sum_{i=1}^{n} {DD}_{i}$$

where $n$ is $DI$.

If we knew the average temperature of every half of the day, then:

$${DD}_{i}=\frac{1}{2}\left( T_{i}^{1}-t_{0} \right)+\frac{1}{2}\left( T_{i}^{2}-t_{0} \right)$$

where $T_{i}^{1}$ is the average temperature for the first half of the $i$-th day and $T_{i}^{2}$ is the average temperature for the second half of the $i$-th day.

We may divide each day into smaller parts. Therefore, the most accurate number of degree-days is equal to the area under the curve defined as follows:

$$r\left( x \right)=\left\{ \begin{matrix} T\left( x \right)-t_{0} & T\left( x \right)>t_{0} \\ 0 & T\left( x \right)\leq t_{0} \end{matrix} \right\}$$

where $T\left( x \right)$ is the temperature observed in the moment $x$ of a year. In other words $T\left( x \right)$ is a continuous function of temperature.

Therefore, a continuous version of the thermal summation method is defined by integral of $r(x)$ function:

$$K=\int_{x_{1}}^{x_{2}} r(x)dx$$

where $x_{1}$ is oviposition, and $x_{2}$ is eclosion. Assuming $\int r\left( x \right)dx=R(x)$ and using Newton-Leibniz formula:

$$K=R\left( x_{2} \right)-R(x_{1})$$

$K$ is therefore equal to the area under the curve $r\left( x \right)$ between the points $x_{1}$ and $x_{2}$.

In practice $x_{1}$ and $x_{2}$ are unknown. The date of cadaver finding ($x_{0}$) is known and $x_{2}=x_{0}-PEI$. We denote$x_{1}$ as $Ov(PEI)$ ($Ov$ from oviposition).

$$K=R\left( x_{0}-PEI \right)-R(Ov(PEI))$$

We obtain:

$$Ov\left( PEI \right)=R^{-1}(R\left( x_{0}-PEI \right)-K)$$

where $R^{-1}(x)$ is an inverted function to $R(x)$. Assuming that the domain of $r(x)$ is defined as one year, $R^{-1}(x)$ returns a moment in which $x$ degree-days have been accumulated since the beginning of a year.

The degree-days are greater than or equal to zero in each day, so $R(x)$ is always a non-decreasing function, irrespective of the $T\left( x \right)$. When $T\left( x \right)\leq0$ (temperatures are less than zero in some period), $R(x)$ is constant in some interval. This means that $R(x)$ may be non-invertible and $R^{-1}(x)$ does not exist. In such cases we assume:

$$\forall x\in(a,b):R\left( x \right)=c=const. \Rightarrow R^{-1}\left( c \right)=a$$

In other words, $c$ degree-days have been accumulated at $a$, and between $a$ and $b$ temperatures were lower than $t_{0}$, so between these moments ($a$ and $b$) no new degree-days have been accumulated.

Because $\min PMI=DI+PEI$, it is possible to find a $PEI$ that provides the least $\min PMI$.

$$\min PMI=x_{0}-Ov\left( PEI \right)$$

The above equation is minimal when $Ov\left( PEI \right)$ is maximal, because $x_{0}$ is a fixed value. As it was mentioned above $R(x)$ is a non-decreasing function, which implies that $R^{-1}(x)$ is also non-decreasing. This means that the maximum value of the function is obtained for the maximum argument (or more precisely the supremum argument, as the constant function has no maximum value and $R$ may be constant in some interval).

Therefore, $argsup\left( Ov\left( PEI \right) \right)=argsup\left( R^{-1}(R\left( x_{0}-PEI \right)-K) \right)=argsup(R\left( x_{0}-PEI \right)-K)$. $K$ is fixed, so it does not influence the supremum argument of the function. We obtain $argsup\left( R\left( x_{0}-PEI \right) \right)=argsup(x_{0}-PEI)$.

$PEI$ cannot be less than zero, so $x_{0}-PEI$ is maximal when $PEI=0$, because $x_{0}$ is fixed.

Concluding, the optimal $PEI$ for the true minimum $PMI$ is zero. This conclusion is general and relevant for all fly species. The fly species (or more precisely fly populations) have specific values of $K$ and $t_{0}$. These factors were considered as fixed during the analysis, which is independent of the specific values of fixed factors. They may change the minimum $PMI$ estimate, but the true minimum $PMI$ will always be reached at $PEI$ of 0.
